# Concurrent validity and reliability of measuring range of motion during the cervical flexion rotation test with a novel digital goniometer

**DOI:** 10.1186/s12891-020-03525-6

**Published:** 2020-08-11

**Authors:** Kerstin Luedtke, Thomas Schoettker-Königer, Toby Hall, Christine Enns, Maike Grassold, Petra Hasselhoff-Styhler, Christian Neulinger, Max Obrocki, Philipp Przyhoda, Axel Schäfer

**Affiliations:** 1 Laboratory of Pain Research, Institute of Physiotherapy and Health Sciences, The Jerzy Kukuczka Academy of Physical Education, Katowice, Poland; 2 Institute of Health Sciences, Academic Physiotherapy, University of Luebeck, Lübeck, Germany; 3 Faculty of Social Work and Health, University of Applied Science and Art (HAWK), Goschentor 1, 31134 Hildesheim, Germany; 4 School of Physiotherapy and Exercise Science, Curtin University, Perth, WA Australia; 5 Faculty of Social Sciences, University of Applied Sciences Bremen, Neustadtswall 30, 28199 Bremen, Germany

**Keywords:** Reliability, Validity, Intra-rater, Inter-rater, Flexion-rotation-test, Neck pain, Headache, Cervical, Zebris, Goniometer

## Abstract

**Background:**

Headache is a common and costly health problem. Although the pathogenesis of headache is heterogeneous, reported contributing factors are dysfunctions of the upper cervical spine. The flexion rotation test (FRT) is a commonly used diagnostic test to detect upper cervical movement impairment. A digital goniometer may support precise measurement of movement impairment in the upper cervical spine. However, its reliability and validity is not assessed, yet. The aim of this study was to investigate the reliability and validity of the digital goniometer compared to an ultrasound-based movement analysis system.

**Methods:**

Two separate cross-sectional studies were conducted using the digital goniometer EasyAngle (Meloq AB, Stockholm, Sweden) for a) investigating the concurrent validity of upper cervical range of motion (ROM) during the FRT and b) determining the inter- and intra-rater reliability in the target population of patients with head and neck pain. Sixty-two participants, 39 with and 23 without head and neck pain, were recruited for the concurrent validity study. For the reliability study, a total of 50 participants were recruited. Intraclass correlation coefficients (ICC) and Bland Altmann plots were used to assess validity and ICC values, Bland Altmann plots as well as Kappa coefficients were used for estimating intra-rater and inter-rater reliability.

**Results:**

Concurrent validity was strong with an ICC (2,1) of 0.97 for ROM to either side (95%CI = 0.95–0.98). Bland Altman Plots revealed a mean difference between measurement systems of 0.5° for the left and 0.11° for the right side.

The inter-rater ICC (2,1) was 0.66 (95%CI 0.47–0.79, *p* <  0.001, SEM 6.6°), indicating good reliability. The limits of agreement were between 10.25° and − 11.89°, the mean difference between both raters was − 0.82°. Intra-rater reliability for the measurement of ROM during the FRT was between 0.96 (ICC 3,1) for rater 1 and 0.94 (ICC 3,1) for rater 2.

**Conclusions:**

The digital goniometer demonstrated strong concurrent validity and good to strong reliability and can be used in clinical practice to accurately determine movement impairment in the upper cervical spine.

**Trial registration:**

German Registry of Clinical Trials DRKS00013051.

## Background

Neck pain and headache are frequent symptoms occurring as separate entities or inter-dependently [1]. Combined symptoms occur in cervicogenic headache (CGH) where a dysfunction of the cervical spine is by definition the source of the headaches. In contrast, in primary headaches such as migraine, neck pain is commonly observed as an associated symptom. It is the topic of an ongoing scientific debate whether cervical nociceptive input might also influence primary headaches [2, 3]. In both cases, the assessment of the upper cervical spine, as a potential contributing factor to either neck pain or headaches is recommended. One of the best evaluated tests for this purpose is the flexion-rotation-test (FRT), which investigates primarily the range of motion (ROM) between the C1 and C2 vertebrae [4–9]. Typically, in standard physiotherapy settings, an eyeballed estimate of ROM is sufficient for clinical purposes [10]. However, this level of measurement may not comply with scientific standards.

One precise method to measure cervical ROM is an ultrasound-based movement analysis system (Zebris GmbH, Isny, Germany) [11, 12]. Although this system offers accurate data, difficulties arise as the system is expensive and requires two physiotherapists for operation, thereby limiting its practicability.

A more practical solution is the cervical ROM device (C-ROM, Platismo Airguide Inc., Buffalo Groove, IL). A systematic review attests that the FRT has a high level of reliability and diagnostic accuracy when this device is used for patients with CGH [13]. Inter-rater reliability has been established in only one previous study: Hall et al. (2008) found that experienced physiotherapists (ICC 0.93 (95%CI 0.87–0.96)) as well as less experienced physiotherapists (ICC 0.84 (95CI% 0.67–0.93)) achieved excellent inter-rater reliability for patients with headache with and without upper cervical dysfunction as well as for headache free controls [8].

A disadvantage of the C-ROM is that patients have to maintain a static posture while the examiner is documenting the values obtained [14]. Clinical experience with the C-ROM further shows that the two inclinometers and the compass system (for the measurement of rotation) do not always offer precise data, since the scale is not always easy to read in certain head positions and the needle of the compass may not always move freely in combined head positions.

A new digital device (*EasyAngle, Meloq AB, Stockholm, Sweden*) might be a practicable and precise alternative to the C-ROM since it is portable, small, easy to handle, not expensive, and stores up to 5 measurements. Its precision is reported by the manufacturing company as +/− 1°. A major advantage over digital goniometers used in previous studies [15, 16] is, that movement can be measured in any one of the three planes without having to realign its position.

The aim of this study was therefore to evaluate the validity as well as intra- and inter-rater reliability of the EasyAngle goniometer during the FRT, in healthy participants as well as patients with headache and/or neck pain.

## Methods

### Study design

Two separate cross sectional studies were conducted to determine the concurrent validity as well as inter- and intra-rater reliability of the EasyAngle goniometer during the FRT.

### Subjects

For the reliability study, a minimum sample size of 50 was required to provide statistically significant information expecting an ICC of 0.75 with a 95% confidence interval of 0.2 and 3 repeated measurements. For the validity study, we recruited participants with and without symptoms to be able to measure a wide variability of range of motion. The calculated sample size was 62 (31 for each group) expecting an ICC of 0.85 with 2 replications and a 95% CI of 0.2.

Participants for the reliability study were recruited from physiotherapy clinics and a dentist clinic in Austria. Participants for the validity study came from the university campus and social media advertising in Bremen/Germany. An information sheet was provided to potential participants explaining general information, procedure, inclusion and exclusion criteria prior to the initial appointment. Inclusion criteria required participants to be aged between 18 and 80 years. Participants for the validity part of the study were either asymptomatic or suffered from headache or neck pain.

For the reliability part of the study, only patients above the age of 18 years suffering from neck pain, headache and/or temporomandibular dysfunction were included, who had been referred for physiotherapy assessment and treatment.

Participants with and without symptoms in both parts of the study were excluded if they had experienced a headache within the 48 h prior to testing, cranial or cervical injury within the last 3 months, a diagnosis of cervical radiculopathy, were pregnant, suffered from osteoporosis, rheumatoid arthritis, or neurological disease. In all aspects of this study, the principles of the „Declaration of Helsinki “[17] was followed at all times. For both parts of the study individual participant populations were recruited. None of the participants received monetary rewards or financial compensation.

### Procedures

After participants provided signed informed consent, a set of questionnaires was completed to characterise the study participants and standardised pre-test screening was conducted to exclude participants with vertebral artery dysfunction or upper cervical instability.

Two physiotherapists with 8 years of clinical experience in treating patients with neck pain were involved in the experiment to evaluate the intra- and inter-rater reliability. For the validity part of the study, one physiotherapist with a clinical experience of 20 years in treating patients with headache and neck pain performed the FRT measurements, a second physiotherapists with 2 years of clinical experience recorded the data. All examiners attended a 45-min training session on the FRT prior to the data collection as well as familiarisation with the different measurement systems.

For the FRT, participants were positioned in supine. The examiner moved the cervical spine into maximum flexion. From this position, the head was rotated to each side. Rotation occurred around an imaginary axis from the vertex of the skull to the central point of the upper cervical spine. The end of range was determined when moderate resistance or pain was noted [4]. The test was aborted in the presence of signs of dizziness and/or nausea.

#### Digital goniometer

**Fig. 1:**
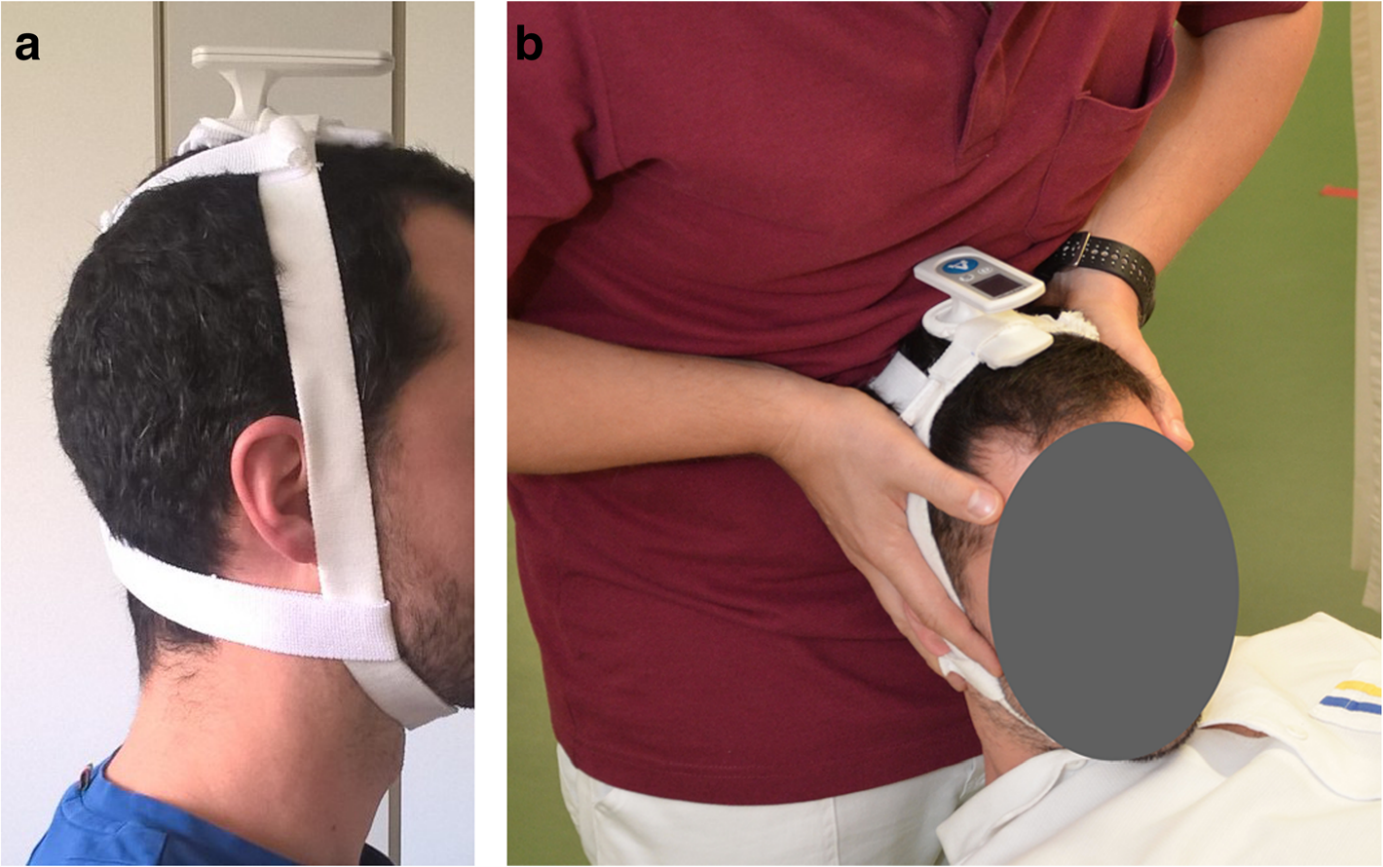
a and b Reliability of the FRT measured with the easyangle. The digital goniometer was fixed via a selfmade elastic band

**Fig. 2:**
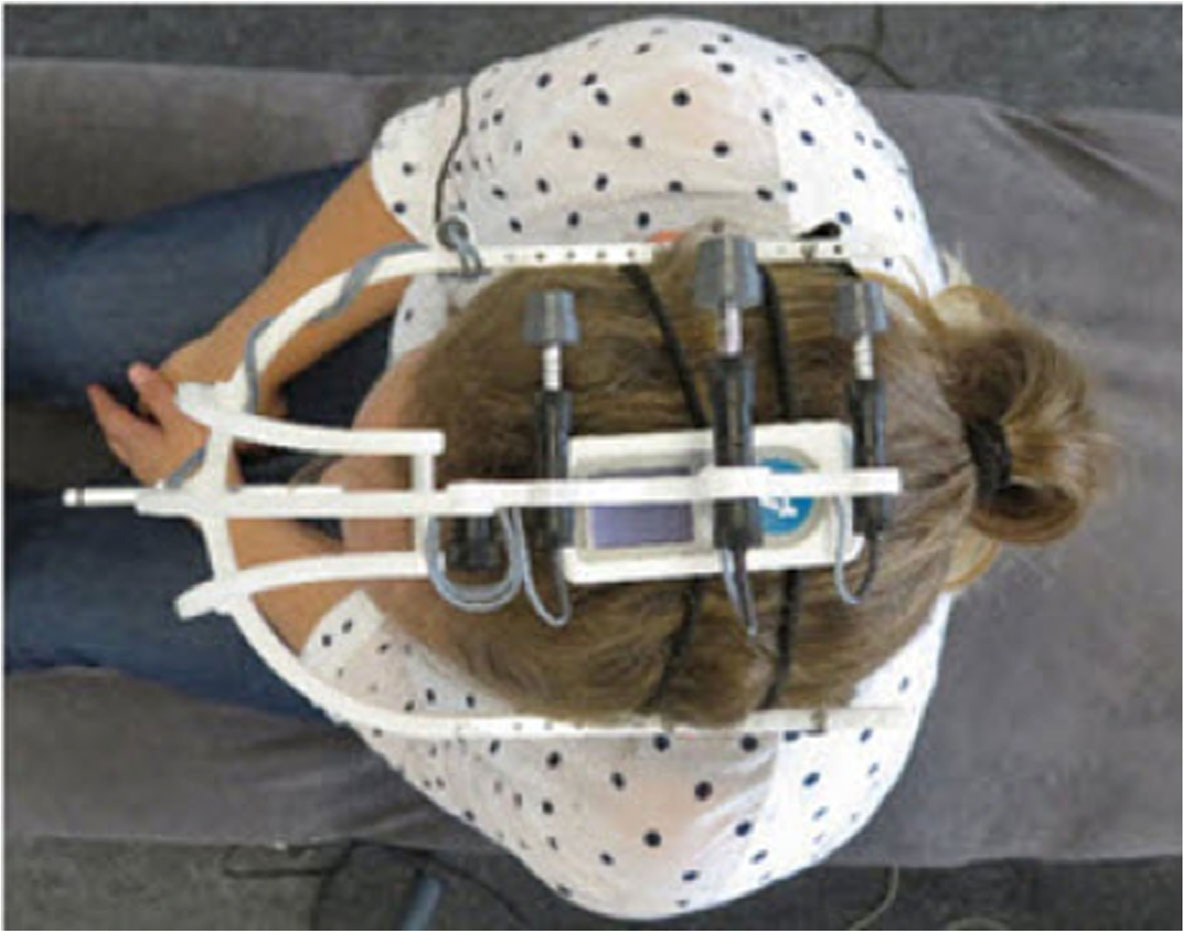
Validity of the FRT. Fixation of FRT and easyangle

The base of the EasyAngle goniometer was positioned on the axis of rotation of the head and fixed with elastic straps (Figs. 1 and 2).

#### Concurrent validity

For the evaluation of validity, the test was simultaneously recorded by an ultrasound based measurement system (Zebris GmbH, Isny, Germany) which includes a helmet with three ultrasonic transmitters. The transmitters were placed in a parallel position on the head of the participant (Fig. 2). The signals from the helmet are detected in real time (50 ms interval with a frequency of 20 Hz) by a sensor positioned at 80 cm distance from the participant. The data is recorded by the WinSpine 2.2 Software for Windows and exported as an ASCII data file. The EasyAngle goniometer was attached to the head of the participant using elastic straps.

During the measurements communication between examiner, subjects and operator of the Zebris system followed standardised procedures. In a pilot phase, five participants were tested to standardise the simultaneous recording with the *Zebris*- and *Easyangle*-systems. These data were not included in the final data set.

Numerous studies have investigated the reliability and validity of the Zebris system to measure cervical range of motion. Dvir et al. [18] found a high correlation (*r* > 0.99) and an average absolute difference between 0.31° and 0.89° in comparison to a digital inclinometer for all primary movements. Strimpakos et al. [19] compared Zebris measurements with x-ray and showed ICC values of 0.88 for flexion and 0.95 for extension, however the sample was small with *n* = 10. The accuracy of the Zebris system for detecting atlanto-occipital movement has also been investigated in an in vitro study indicating an error of less than 0.2° with each of the three motion components [20].

In a previous study [21] the SEM for rotation ROM during the FRT was 2.3 (left) and 3.1 (right) corresponding to a SDD of 6.3° (left) and 8.5° (right), which is lower than the SEM of 7.88 and SDD of 21.83 reported by Cagnie et al. [21] for active rotation. Mannion et al. [11] found substantial differences between measurements of the Zebris system compared to a CA6000 Spine Motion Analyser in measuring cervical rotation in flexion. However, it has to be taken into account that the flexion rotation test in their study was performed actively in sitting position. Mannion et al. [11] state that performing the test was difficult for the participants, possibly leading to a less reliable performance for this test. This is in contrast to our study where the test was carried out passively in supine lying, which may result in a more stable and reliable performance.

#### Intra- and inter-rater reliability

Each participant was assessed using the EasyAngle goniometer by two examiners in a randomised order at two occasions. On each occasion, three consecutive measurements were taken with a 10-min time interval between each trial*.* The measurements from the consecutive three measurements were used to assess intra-rater reliability. For inter-rater reliability we calculated the mean values of the three measurements on each occasion. For the inter-rater reliability, the examiner was blinded towards the result of the second examiner. For the intra-rater reliability, the examiner was blinded towards the results of the previous measurement. This was achieved by covering the display of the EasyAngle goniometer during the measurement. Measurement data were extracted by a second therapist. An independent examiner documented ROM, the limiting criterion (pain or resistance), and the location if any of the pain response (neck, head, face) during the test. This person was otherwise not involved in any other study procedures.

#### Questionnaires and assessments

Additional information about the participants was collected using questionnaires. These included demographic data (age and sex) and a checklist confirming inclusion- and exclusion criteria. Symptomatic subjects were requested to fill out the neck disability index (NDI) [22] and the Migraine Disability Assessment (MIDAS) to determine disease specific disability levels [23]. If patients reported temporomandibular symptoms, the Conti Anamnestic Questionnaire CMD was used [24].

To screen for upper cervical instability we performed the sharp purser test [25], test of the transverse ligament [26] and a stress test of the alar ligament [27]. Additionally, all subjects performed the FRT actively in sitting before testing. Screening for vertebral artery dysfunction was performed in sitting, according to APA recommendations [28].

### Statistical analysis

Mean, standard deviation, minimum and maximum values from the three repetitions were calculated for each examiner and for each participant as well as for each measurement system.

Intra-rater and inter-rater reliability as well as concurrent validity were determined by calculating the ICC with their 95% confidence intervals (CI). A two-way random-effect model based on a single rating and absolute agreement was used to assess inter-rater reliability and concurrent validity (ICC 2,1) [29]. A two-way mixed effect-model based on consitency and single rating assessed the intra-rater reliability for either rater (ICC 3,1) [30]. Furthermore the standard error of measurement (SEM) and the smallest detectable difference (SDD) were given. The SEM was estimated using the formula SEM_agreement_ = √(σ^2^_error_ + σ^2^_residual_). The SDD as calculated using the formula SDD = 1.96 × √2 × SEM. To estimate the inter-rater reliability for the limiting factors of the FRT (pain vs resistance) Fleiss‘Kappa (κ) was used.

The comparability between the zebris system and the easyangle and between the two raters was visualised by Bland-Altman-plots [31] and scatter plots.

For the interpretation of the ICC, < 0.40 was regarded as insufficient and > 0.75 as almost perfect agreement [32]. The results from Fleiss’ Kappa were interpreted using the recommendations by Landis & Koch with 1–0.81 indicating excellent agreement [33]. All statistical analyses were carried out using STATA 15.1 [34]..

## Results

Table 1 shows the demographic details, presence of neck pain and headache and disability levels for both the validity and reliability study.

**Table 1:** Characteristics of the participants (n = number of participants; f = female; m = mean; SD = standard deviation)

	Validity study (***n*** = 62)	Reliability study (***n*** = 50)
Sex f (n (%))	38 (61)	33 (66)
Age (m (SD))	41.94 (16.27)	42.78 (12.41)
Headache and / or neck pain (n (%))	39 (62.90)	–
Headache	–	30 (60)
Neck pain	–	38 (76)
**NDI** (n (%))		
1 (1–8 points) mild symptoms	13 (20.97)	12 (29.27)
2 (9–39 points) moderate symptoms	26 (41.94)	27 (65.85)
3 (40–100 points) severe symptoms	0 (0)	2 (4.88)
**Midas** (n (%))		
1 little impairment	22 (35.48)	11 (35.48)
2 minor impairment	6 (9.68)	6 (19.35)
3 moderate impairment	4 (6.45)	8 (25.81)
4 severe impairment	7 (11.29)	6 (19.35)

### Concurrent validity

Data for mean values and standard deviations for the ultrasound based system and the goniometer measurements are presented in Table 2.

**Table 2:** Mean and standard deviation of individual and average ultrasound based (Zebris) and digital goniometer (easyangle) measurements. *(*n = number of participants; SD = standard deviation)

	n	mean°	SD°		mean °	SD °
zebris_left_1	62	42.43	9.43	easyangle_left_1	42.20	8.64
zebris_left_2	62	43.80	8.38	easyangle_left_2	43.13	8.54
zebris_left_3	62	44.80	8.84	easyangle_left_3	44.13	9.19
mean_zebris_left	62	43.68	8.39	mean_easyangle_left	43.15	8.30
zebris_right_1	62	41.14	9.30	easyangle_right_1	40.95	8.71
zebris_right_2	62	42.24	9.59	easyangle_right_2	41.82	9.59
zebris_right_3	62	43.29	9.60	easyangle_right_3	43.60	8.67
mean_zebris_right	62	42.21	9.27	mean_easyangle_right	42.32	8.25

**Fig. 3:**
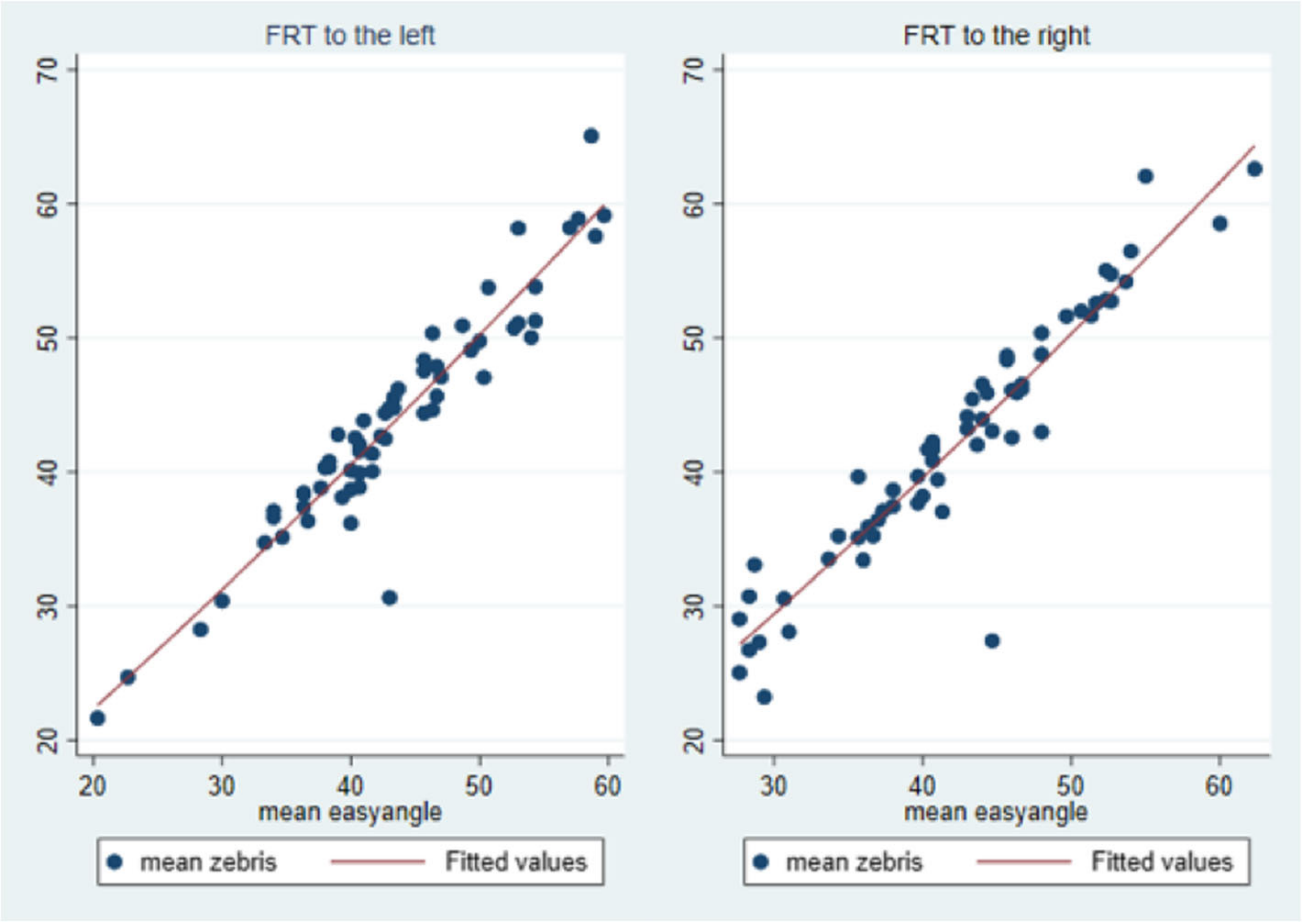
Scatterplot showing the mean values measured with easyangle and zebris

ICC-values and 95% CIs are presented in Table 3 indicating the concurrent validity between the goniometer and the ultrasound based measurement system. For visualisation of the relationship, scatterplots are provided (Fig. 3).

**Table 3:** ICC (2,1) of digital goniometer and Zebris for the left and right rotation. (ICC (2,1) = Intraclass Correlation Coefficient; CI = Confidence Interval*)*

	ICC	[95%CI]	
**Left rotation**			
Individual	.95	.92	.97
Average	.97	.96	.98
**Right rotation**			
Individual	.94	.90	.96
Average	.97	.95	.98

**Fig. 4:**
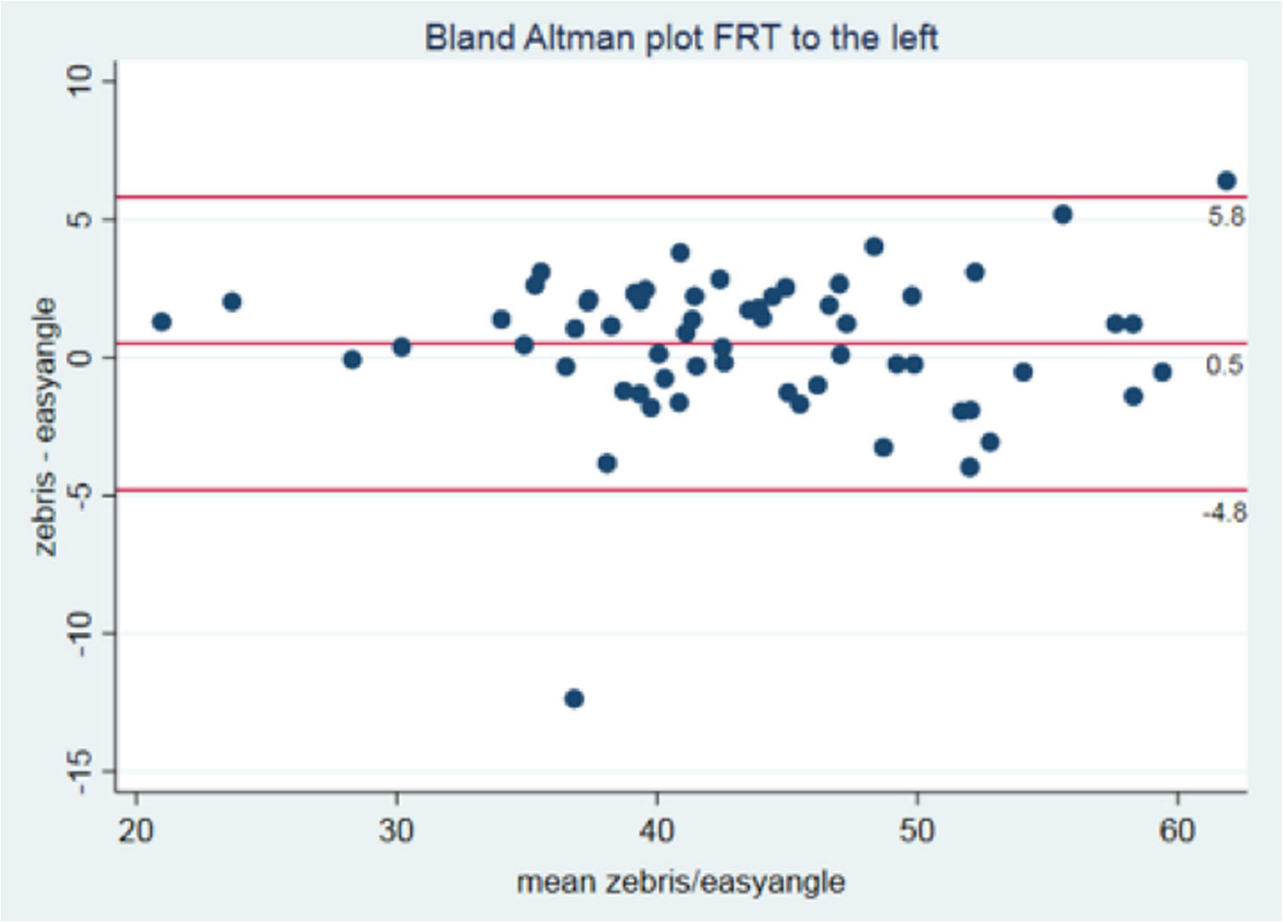
Bland Altman Plot for left rotation

**Fig. 5:**
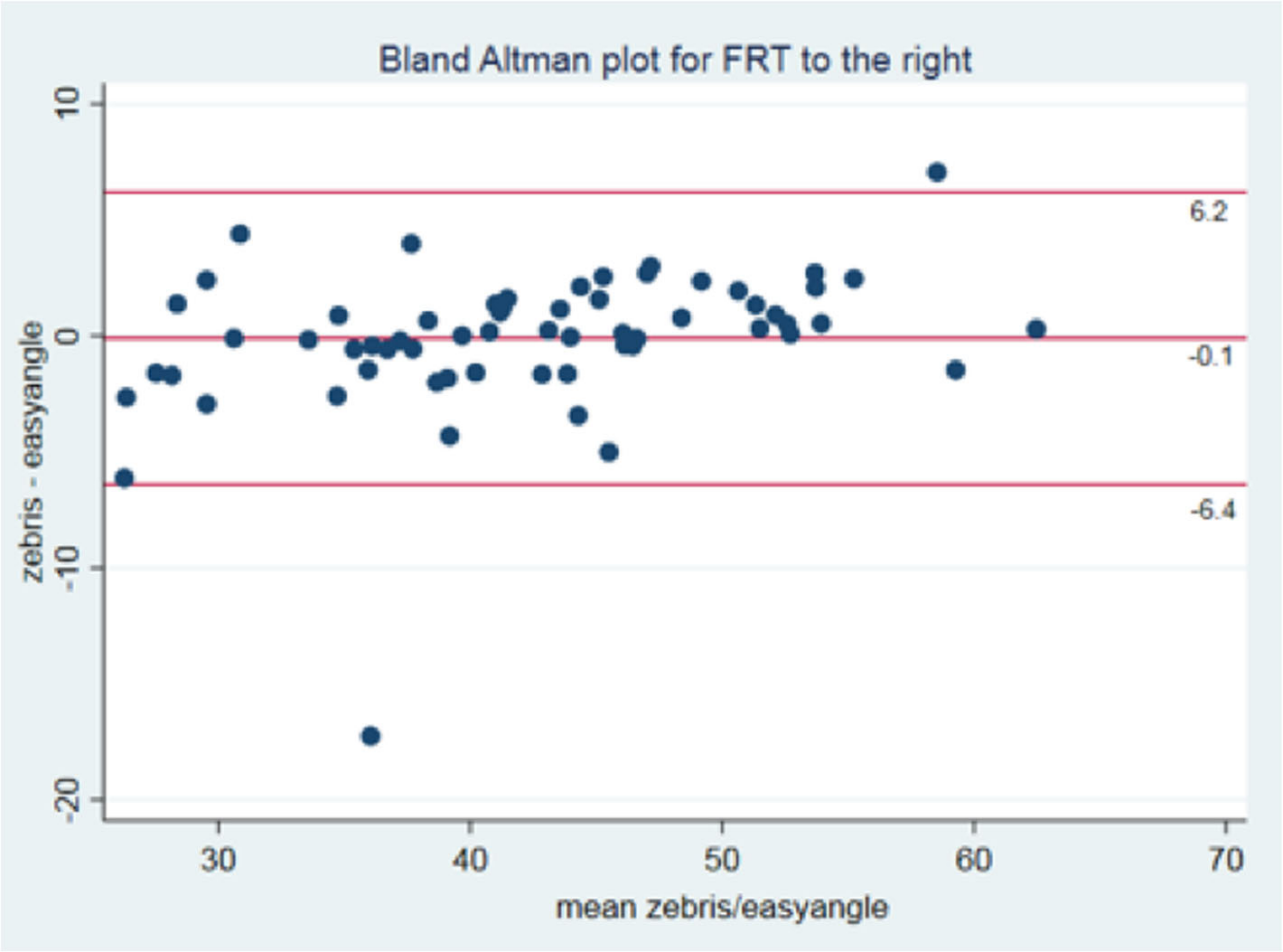
Bland Altman Plot for right rotation

The limits of agreement for the left rotation were between − 4.80° to 5.85°. The mean difference was 0.53° (95%CI -0.15 - 1.29). The limits of agreement for the right rotation were between − 6.39° to 6.17°. The mean difference was − 0.11° (95%CI -0.91 - 0.69) (Figs. 4 and 5).

### Reliability

Intra-rater reliability data for the measurement of ROM during the FRT determined by the EasyAngle goniometer are displayed in Table 4.

**Table 4:** Intra-rater reliability. (ICC Type (3,1) = two way mixed-effects model, absolute agreement, individual; Number of targets 50; number of ratings 3; all ICC values are significant with *p* < 0.0001)

FRT	Examiner 1			Examiner 2		
	ICC (3,1)	SEM	SDD	ICC (3,1)	SEM	SDD
Right	0.954 (95%CI 0.89–0.97)	1.56°	4.32°	0.924 (95%CI 0.88–0.95)	1.8°	4.99°
Left	0.955 (95%CI 0.93–0.97)	1.35°	3.74°	0.94 (95%CI 0.9–0.96)	1.68°	4.66°
Total	0.96 (95%CI 0.94–0.98)	2.37°	6.57°	0.936 (95%CI 0.9–0.96)	2.85°	7.90°

In terms of inter-rater reliability, examiner 1 showed a smaller range between average minimum and maximum values compared to examiner 2 (Table 5). The total FRT maximum right to maximum left showed an agreement of 0.66 (95%CI 0.47–0.79; *p* <  0.001; SEM 6.6°) (Table 5).

**Table 5:** Inter-rater reliability. (MW = mean, SD = Standard deviation, ICC intraclass correlation Type (2,1) absolute agreement. SEM measurement error)

FRT	Mean, SD, and range						Inter-Rater Reliability		
	Examiner 1			Examiner 2					
	MW	SD	Min/Max°	MW	SD	Min/Max°	ICC (95%CI)	p	SEM
Right	43.49°	7.18°	28.67/59.33	43.92°	6.36°	25/58	0.60 (0.39–0.75)	< 0.001	4.2°
Left	46.03°	6.31°	30.33/59	45.65°	6.73°	32.33/64.33	0.72 (0.56–0.83)	< 0.001	3.4°
Total	89.53	11.61	69.66/115.33	89.57	11.10	67.66/116	0.66 (0.47–0.79)	< 0.001	6.6°

The limits of agreement for rotation to the left are between + 9.8° and − 9.02° The mean difference is 0.39°. The limits of agreement for rotation to the right are between + 11.52° and − 12.39°. The mean difference is 0.43°.

Evaluation of the limiting factor (stiffness or pain) showed that during FRT to the right, examiner 1 reported stiffness 36 times (72%) and pain 14 times (28%), 13 of which were in the neck and once headache. Examiner 2 reported stiffness 37 times and pain 13 pain (12 times neck pain and once headache). Temporomandibular pain was not reported during the assessments. The Kappa value for the limiting factor for FRT to the right was κ = 0.85 (*p* < 0.001). Results are shown in Table 6.

**Table 6:** Agreement on limiting factor between examiners

	Limiting factor				Kappa	***p***
	Stiffness	Neck pain	Headache	Jaw pain		
**FRT right**						
Examiner 1	36	13	1	0	0.85	< 0.001
Examiner 2	37	12	1	0		
**FRT left**						
Examiner 1	39	10	0	1	0.89	< 0.001
Examiner 2	37	12	0	1		

FRT to the left was limited 39 times by stiffness (examiner 1) and 11 times by pain (10 of which were in the neck, 1 in the jaw). Examiner 2 reported stiffness 37 and pain 13 times (12 neck of which were in the neck, 1 in the jaw). Headache was not reported as a limiting symptom on any occasion. The kappa value for the limiting factor during FRT to the left was κ = 0.89 (*p* < 0.001) (Table 6).

No adverse events (e.g. dizziness or nausea) were reported during any of the tests.

## Discussion

Concurrent validity for the novel EasyAngle goniometer, when measuring ROM during the FRT, was very good with an ICC (2,1) of 0.97 for movement to the left or the right, suggesting that this goniometer is a valid instrument to measure ROM during the FRT. The Bland Altman Plots (Figs. 4 and 5) support this result indicating a mean difference between both measurement systems of only 0.5° for the left side and 0.11° for the right side. Based on this finding, measurements taken by this EasyAngle goniometer can be regarded as accurate.

Our results are based on a sufficient sample size of participants with a wide age distribution (19–76 years), mix of genders (female 61%), asymptomatic and symptomatic (62.90%) participants and subjects with a diverse ROM during the FRT (20.33–65.08°). Average values identified for the FRT in both, symptomatic and asymptomatic subjects, reflects those reported in the literature [9]. In a previous study [10] the SEM for rotation ROM during the FRT was 2.3 (left) and 3.1 (right) corresponding to an SDD of 6.3° (left) and 8.5° (right), which is much lower than the SEM and SDD reported by Cagnie et al. [21] for active rotation. Hence, these results can be applied confidently to a wide range of clinical presentations.

The inter-rater reliability was good and is comparable to reliability data reported by Hall et al., who measured the FRT with a C-ROM device [8]. Interestingly, in the present study the ICC value (2,1) for the FRT was greater for range to the left 0.72 (95%CI 0.56–0.83) than to the right 0.60 (95%CI 0.39–0.75). The ICC value for range to the right is just above the cut off value of 0.60 which is regarded as fair agreement [35]. The corresponding SDD were 11.64° for FRT to the left and 9.42° for FRT to the right, indicating that differences between measurements should exceed these values in order to differentiate real change from measurement error, when two examiners measure at different time points.

The intra-rater reliability was very good, with ICC (3,1) values exceeding 0.92. Furthermore, corresponding SDD values for measurements of the FRT to the left and right were < 5.0°, indicating more precise measurements when only one examiner took the measurement. However, in the interpretation of these results, SDD measures of the concurrent standard Zebris need to be considered. SDDs for rotation ROM during the FRT was 6.3° (left) and 8.5° (right) [2]. We recognise that the Zebris system, although used as a reference standard, is not free from measurement error. To mitigate against this we performed the measurement of the FRT with both systems simultaneously, to reduce handling and other errors. Furthermore, in the analysis we used Bland Altmann plots to chart the difference between both measurements and the mean of both values. The Bland Altmann plot shows that the limits of agreement varies between 6.2° and − 6.4° and the mean difference is 0.5° and − 0.1° for the FRT to the left and right respectively.

Although eyeballed estimation has been shown to be a valid form of measurement to determine a positive or negative FRT [2], measuring the exact range of motion may be useful to distinguish different types of headache. For example, Hall et al. [5] identified a cut-off point for a positive FRT to be 30° of rotation or less, when attempting to distinguish a patient with CGH from migraine without aura or mixed headache forms. This is less than the cut-off value of 32° reported by Ogince et al. [9]. In that study the groups were more diverse and included patients with CGH or migraine with aura, as well as asymptomatic controls. Hence a larger reduction of rotation was required to differentiate migraine from cervicogenic headache, or both from asymptomatic controls. Confirmation of this finding is shown in the study by Oliveira Souza et al. (2019). In that study patients with episodic migraine had a mean range of 33° for the FRT to the right and 32° for the FRT to the left [36]. A further example when an accurate measurement of ROM during the FRT is needed, is when the test is used as an outcome measure. A previous study identified that limited rotation during the FRT is strongly correlated with an index of CGH symptoms. This index is comprised of a combination of headache frequency, intensity and duration, with approximately 50% of the variance in range of motion explained by the headache index [5]. Using a simple but accurate tool is a helpful addition to the clinical physiotherapy setting, as the EasyAngle goniometer is small, portable and easy to use. Future research should aim to determine the sensitivity to change and clinically meaningful differences in ROM determined by the FRT in longitudinal studies.

## Limitations

The following limitations need to be taken into account. First of all, the validity and accuracy of the concurrent standard Zebris, especially in detecting upper cervical rotation needs to be considered. While inter- and intra-rater reliability appear to be very good, with acceptable SDD values for upper cervical ROM during the FRT, so far a robust comparison against gold standard radiology has not been conducted for upper cervical ROM during the FRT in vivo.

Secondly, repeated measurements may increase pain sensitivity during the FRT, potentially leading to a greater within-subject variation of upper cervical ROM for those occasions where pain was the criteria to stop the FRT. Furthermore, multiple test-stop criteria (pain, firm resistance) add to the complexity of the FRT test, potentially limiting reproducibility. However, interrater reliability for test-stop criteria was good with kappa values between 0.85 and 0.89. Nevertheless, increased pain sensitivity and multiple test-stop criteria may have potentially deflated ICC values for reliability.

## Conclusion

The EasyAngle digital goniometer has been shown to have very good concurrent validity and good reliability and may aid clinicians to accurately determine movement restriction in the upper cervical spine during the FRT.

## Data Availability

The datasets used and/or analysed during the current study are available from the corresponding author on reasonable request.

## Abbreviations

CGH: Cervicogenic headache
CI: Confidence interval
C-ROM: Cervical range of motion
FRT: Flexion-rotation test
ICC: Intraclass correlation coefficient
MIDAS: Migraine disability assessment scale
NDI: Neck disability index
ROM: Range of motion
SD: Standard deviation
SDD: Smallest detectable difference
SEM: Standard error of measurement
