# Correction to: Concurrent validity and reliability of measuring range of motion during the cervical flexion rotation test with a novel digital goniometer

**DOI:** 10.1186/s12891-020-03633-3

**Published:** 2020-09-21

**Authors:** Kerstin Luedtke, Thomas Schoettker-Königer, Toby Hall, Christine Reimer, Maike Grassold, Petra Hasselhoff-Styhler, Christian Neulinger, Max Obrocki, Philipp Przyhoda, Axel Schäfer

**Affiliations:** 1 Laboratory of Pain Research, Institute of Physiotherapy and Health Sciences, The Jerzy Kukuczka Academy of Physical Education, Katowice, Poland; 2 Institute of Health Sciences, Academic Physiotherapy, University of Luebeck, Lübeck, Germany; 3 Faculty of Social Work and Health, University of Applied Science and Art (HAWK), Goschentor 1, 31134 Hildesheim, Germany; 4 School of Physiotherapy and Exercise Science, Curtin University, Perth, WA Australia; 5 Faculty of Social Sciences, University of Applied Sciences Bremen, Neustadtswall 30, 28199 Bremen, Germany

**Correction to: BMC Musculoskelet Disord 21, 535 (2020)**

**https://doi.org/10.1186/s12891-020-03525-6**

Following publication of the original article [1], the authors noticed that one of the co-authors’ name is incorrect. The author Christine Enns should be changed to Christine Reimer. The original article [1] has been updated.
